# Transient acute kidney injury following femoral fracture in mice: the role of sympathetic activation

**DOI:** 10.1302/2046-3758.158.BJR-2025-0760.R1

**Published:** 2026-08-05

**Authors:** Katharina Oßwald, Hannah Herbst, Sandra Dieterich, Elizabeth Rosado Balmayor, Anita Ignatius, Markus Huber-Lang, Melanie Haffner-Luntzer, Rebecca Halbgebauer

**Affiliations:** 1 Institute of Clinical and Experimental Trauma Immunology, Ulm University Medical Center, Ulm, Germany; 2 Institute of Orthopaedic Research and Biomechanics, Ulm University Medical Center, Ulm, Germany; 3 Experimental Orthopaedics and Trauma Surgery, Department of Orthopaedics, Trauma and Reconstructive Surgery, RWTH Aachen University Hospital, Aachen, Germany

**Keywords:** Femoral shaft fracture, External fixation, Trauma-related acute kidney injury, Adrenoceptor blockade, α-adrenergic signalling, acute kidney injury, Femoral fractures, orthopaedic trauma, femoral shaft fractures, Gene expression, fibrosis, inflammation, blood, high-energy trauma, fracture healing

## Abstract

**Aims:**

Femoral shaft fractures are commonly associated with high-energy trauma, and affect individuals of all ages. Kidney dysfunction frequently complicates fracture healing, and trauma-related acute kidney injury (TRAKI) elevates the risk of adverse outcomes. The mechanisms of post-fracture kidney injury, particularly the involvement of sympathetic activation, remain poorly understood. We aimed to define the impact of femoral fracture on the kidney and investigate mechanistically the potential contribution of sympathetic activation.

**Methods:**

A total of 46 female C57BL/6J mice were subjected to femoral shaft fracture and external fixation, and received daily treatment for three days with either phentolamine, propranolol, or butoxamine. They were analyzed early (at day 1) and late at day 21 post-fracture. Renal function was assessed via blood urea nitrogen measurement, while kidney injury was evaluated using histology and gene expression analysis.

**Results:**

While renal function remained within the physiological range, animals with fracture revealed signs of kidney damage one day after fracture, reflected by increased expression of kidney damage markers and oxidative stress indicators and histopathological changes. Cellular inflammation and proliferation were induced upon fracture but did not cause long-term fibrosis. By 21 days, these effects were no longer detectable, suggesting transient TRAKI. Adrenergic receptor (AR) blockade experiments clearly indicated a sympathetic contribution to this temporal renal response. α-AR-mediated effects in particular modulated the kidneys’ response to oxidative stress, as phentolamine treatment reduced early oxidative stress markers. Furthermore, immune activation was only detected upon propranolol application. However, none of the treatments provided a sustained protective effect. Rather, a mixed outcome was observed in different aspects of TRAKI.

**Conclusion:**

Taken together, femoral fractures in mice can induce transient mild kidney injury, local cellular damage, and inflammation, while maintaining renal function. Adrenergic signalling appears to contribute to TRAKI development, which warrants further mechanistic investigation.

Cite this article: *Bone Joint Res* 2026;15(8):961–972.

## Article focus

- Femoral shaft fractures are common high-energy injuries of the musculoskeletal system and represent a major public health concern.
- Systemic complications such as trauma-related acute kidney injury (TRAKI) are known to adversely affect fracture healing. However, their mechanistic basis remains poorly understood.
- This study aimed to characterize the progression of TRAKI following femoral shaft fracture, and to evaluate the role of sympathetic activation through targeted adrenergic receptor blockade.

## Key messages

- Femoral shaft fractures in mice induce transient mild kidney injury with local cellular damage and inflammation, while maintaining renal function.
- Adrenergic (AR) signalling appears to contribute to TRAKI development, while receptor blockade does not provide a sustained protective effect.
- α-AR-mediated effects modulate the kidneys’ response to oxidative stress, as phentolamine treatment reduced early oxidative stress markers.

## Strengths and limitations

- For the first time, this study demonstrates that α- and β-AR pathways contribute distinctly to different aspects of post-fracture kidney injury, providing mechanistic insight into the complex role of sympathetic regulation.
- The murine fracture model used induces relatively limited tissue injury and mild subsequent TRAKI, which may underestimate the impact of AR receptor blockade.

## Introduction

Physical trauma is a persistent potential danger in our daily life. Bone fractures are the most common injuries of the musculoskeletal system and represent a major public health concern, with femoral fractures exhibiting a high prevalence.^1,2^ Femoral shaft fractures, in particular, are frequently associated with high-energy trauma as the shaft’s biomechanical characteristics seem to absorb substantial impact forces that exceed the bone’s structural strength resulting in fracture.^3^ The reported annual incidence ranges from 10 to 21 per 100,000 patients.^4-6^ While contemporary orthopaedic research has primarily focused on fracture fixation techniques,^7-9^ biomechanical stability, and associated complications such as nonunion or periprosthetic fracture,^10-12^ the systemic consequences of fracture and stabilization remain incompletely understood. High-energy long-bone trauma represents a profound systemic insult through soft-tissue damage, inflammatory activation, and potential muscle breakdown, which may contribute to remote organ dysfunction, including acute kidney injury (AKI). In patients with femoral shaft fractures, an incidence of 6.1% for AKI was reported during their hospital stay.^13^ Moreover, a recent study found that 31.3% of orthopaedic trauma patients undergoing open reduction and internal fixation of femoral fractures developed AKI postoperatively.^14^ This form of AKI has been defined as trauma-related AKI (TRAKI), and the pathophysiology behind it is a cumulation of various trauma-associated drivers, which together lead to kidney damage and a decline in function.^15^ These include the accumulation and deposition of endogenous tissue debris (e.g. from muscle), hypoxic conditions, microcirculation disorders, as well as exposure to pathogens and toxins, all of which have pro-inflammatory and damaging effects on kidney.^15^ In the clinical setting, mortality after femoral fracture was demonstrated to be 3.2-times higher in patients with TRAKI compared to those without. Furthermore, TRAKI-related death seems to correlate with the increase of the patients’ age and the time until surgical intervention takes place.^13^ Therefore, special attention should be given during peritraumatic care to protect the kidney, e.g. by increased monitoring; avoidance of nephrotoxic contrast agents, antibiotics, and drugs;^16^ and care for adequate haemodynamics and fluid balance.

In this context, catecholamines like norepinephrine (NE) are frequently administered to patients in hospitals to stabilize circulation.^17^ NE is the key effector of the sympathetic nervous system, which is immediately activated during trauma and injury.^18^ This stress response aims to restore cardiovascular and hemodynamic stability, mobilize stored energy to meet the enhanced metabolic needs, and ensure the maintenance of immune competence and tissue repair.^18^ Furthermore, sympathetic efferents interact directly with renal vasculature, tubules, and juxtaglomerular granular cells. Tissue injury, hypoxia, and other trauma-related drivers activate the interactive sympathetic network.^19^ The consequence of this activation is an even higher NE generation,^20,21^ triggering a catecholamine-mediated vasoconstrictive response in the kidneys.^15^ This vasoconstriction reduces renal blood flow (RBF), subsequently leading to a decrease in glomerular filtration rate (GFR) and urine output. The effects of NE are mediated through binding to adrenergic receptors (AR). The AR presence in various nephron segments was demonstrated previously.^22,23^ Thus, we hypothesized that sympathetic activation following trauma contributes to the pathogenesis of TRAKI by modulating the temporospatial renal response, and that AR blockade may confer protective effects on the kidney and improves clinical outcome. Therefore, we investigated the effects of the non-selective β-adrenergic antagonist propranolol, the selective β_2_-adrenergic antagonist butoxamine, and the non-selective α-adrenergic antagonist phentolamine, especially considering that these drugs are often already taken on a regular base before experiencing trauma.^24,25^

Overall, this study aims to elucidate not only the extent to which experimental femoral fracture induces kidney damage but also to give insights into the therapeutic potential of AR blockade in mitigating post-traumatic renal injury.

## Methods

### Animals

For this study, 46 female C57BL/6J wild-type mice (Charles River Laboratories, Germany) at the age of 12 weeks and a weight of 21 g to 23 g were used. Animals had free access to water, food, and nesting material, and were maintained under a 12/12-hour light–dark cycle. Acclimated for at least two weeks after arrival in cages of four, the mice were randomly divided into four fracture groups (N = 10 per group) that received AR-blocker/vehicle treatment and a control group (N = 6). The sample size was calculated based on a priori power analysis using G*Power, with an effect size of 0.66, assuming a one-way analysis of variance (ANOVA), a significance level of 5%, and a power of 80% with the bone volume per tissue volume (BV/TV) as primary outcome. Whereas a separate animal cohort (not included in this study) underwent bilateral ovariectomy as described previously,^19^ all mice in the present experiment underwent a sham operation, serving as controls for the ovariectomized cohort in the other study. Only the operator was aware of the allocation of mice to the different treatment groups during the experiment. For pain relief, the animals received tramadol hydrochloride (Tramal drops) at a concentration of 0.1 mg/ml via the drinking water for a period of one day preoperatively and three days postoperatively for all surgical procedures. Immediately after induction of anaesthesia, the mice received a single subcutaneous injection of Tramal (25 mg/kg). Euthanasia was performed painlessly by an overdose of isoflurane.

### Femur osteotomy and adrenergic blockade

Femur osteotomy was performed four weeks after sham operation according to an established protocol.^26^ The mice were anaesthetized and received a subcutaneous injection with AR blockers immediately before femur osteotomy and once daily for the first three days after fracture surgery. Injections were necessary to ensure that all mice received the same daily dose of the blockers. The maximum injection volume was 100 µl and the respective blocker concentration was as follows: α-blocker phentolamine (5 mg/kg) (Abcam, UK), β-blocker propranolol (5 mg/kg) (Supelco, Germany), β_2_-blocker butoxamine (4.2 mg/kg) (Santa Cruz Biotechnology, USA), or NaCl (Sigma-Aldrich, USA) as vehicle. After lateral incision of the skin and preparation of the fascia between the musculus biceps femoris and the musculus lateralis of the musculus quadriceps femoris, the femur of the right hind limb was exposed. Subsequently, the external fixator was attached to the craniolateral side of the femur using four mini-Schanz screws. The femur was osteotomized between the two innermost screws of the external fixator using a Gigli wire saw, before suturing muscle and skin. The fractured mice were euthanized at one day (N = 6 per group) or 21 days (N = 4 per group) after fracture surgery with an overdose of isoflurane. Kidneys were harvested for histological analysis or snap-frozen in liquid nitrogen and stored at -80 °C to be analyzed via quantitative polymerase chain reaction (qPCR). Plasma samples were collected to measure blood urea nitrogen levels. Outcome measures were collected under blinded conditions, and all personnel were deblinded only for the final analysis. We have adhered to the ARRIVE guidelines and have included the ARRIVE checklist as Supplementary Material.

### Histological analyses

Mouse kidneys were fixed in 3.7% formaldehyde (Otto Fischar GmbH & Co. KG, Germany) in phosphate-buffered saline (PBS) immediately after harvesting and later dehydrated with ethanol and xylene. Tissues were embedded in paraffin and cut in 4 µm sections.

Periodic acid-Schiff (PAS) staining was performed using PAS staining kit (Sigma Aldrich) according to the manufacturer’s instructions. Nuclei were stained with Gill’s haematoxylin III (Morphisto, Germany) and afterwards mounted with Neo-Mount (Sigma-Aldrich) and glass-covered for long-term preservation.

For immunohistochemical staining (IHC) of the adrenergic receptors, sections were deparaffinized, rehydrated, and boiled in sodium citrate buffer (pH 6.0, Adrb1 and Adrb2) or Tris-EDTA buffer (pH 9.0, Adra1a and Adra2b) for epitope retrieval. After blocking with 10% goat serum (Jackson Laboratories, USA) for 30 minutes, the sections were washed in tris-buffered saline (TBS), followed by incubation with the following primary antibodies (according to Supplementary Table 1): Anti-Adra1a (Thermo Fisher Scientific, USA), Anti-Adra2b (Proteintech, Germany), Anti-Adrb1 (Thermo Fisher Scientific), and Anti-Adrb2 (Abcam). After washing, the primary antibodies were detected using a 1:100 alkaline-phosphatase conjugated goat anti-rabbit antibody (Jackson Laboratories) for 30 minutes at room temperature and stained with a red chromogen (Dako REAL Detection System, Alkaline Phosphatase/RED; Agilent Technologies, USA). Nuclei were stained for one minute with Mayer’s hemalum solution (Sigma-Aldrich). Stained sections were dehydrated in ethanol and xylene and mounted with Neo-Mount (Sigma-Aldrich). Quantification of the staining intensity was performed by examining three random fields of view (100× magnification) per animal, using the Axio Imager M2 microscope equipped with an AxioCam503 Colour camera (both Zeiss, Germany). The intensity sum of channel ‘bright red’ was analyzed for each image with help of the ZEN software (Zeiss). Afterwards, the mean intensity for each animal was calculated.

For visualization of connective tissue in the kidney, trichromic Masson-Goldner staining was performed using Masson-Goldner staining kit (Sigma-Aldrich) and a modified version of the respective protocol. Briefly, sections were deparaffinized and rehydrated in xylene and ethanol before incubating them at 37 °C for 30 minutes in prewarmed Bouin solution (Morphisto). Sections were then incubated for seven minutes in a 1:1 mixture of the two solutions of Weigert′s iron haematoxylin kit (Sigma-Aldrich). The incubation times of the following three staining steps were optimized as follows: Reagent 1, seven minutes; Reagent 2, three minutes; Reagent 3, five minutes. At the end, stained sections were dehydrated in ethanol and xylene, mounted with Neo-Mount (Sigma-Aldrich) and glass covered for long-term preservation.

To check for senescent cells in the kidney after trauma, histochemical staining for beta-galactosidase was performed using the Senescence Cells histochemical staining kit (Sigma-Aldrich). Then, 8 µm thick cryo-sections were stained according to the manufacturer’s protocol for 11 hours at 37°C and then mounted with VECTASHIELD Antifade Mounting Medium (Vector Laboratories, USA).

### Histopathological scoring of the renal tubular injury

To evaluate histomorphological changes in the proximal tubule following trauma, PAS-stained sections were screened for four different injury criteria: 1) tubular cell sloughing, 2) loss of the brush border, 3) tubular dilatation, and 4) tubular cell vacuolization.^27-29^ Representative examples of each type of injury are shown in Supplementary Figure 1A. Damage was scored on a scale from 0 to 3, with 0 indicating a physiological state and 3 representing extensive damage and multiple lesions (Supplementary Table 2). The scoring results are summarized in Supplementary Figures 1B–E. Blinded evaluation was performed by examining 15 random fields of view (400× magnification) per animal, with the same setup as for the IHC staining. For each sample, the mean score for each injury criterion across the 15 images was calculated and subsequently combined into a total injury score for comparative analysis.

### Blood urea nitrogen measurement

Measurement of the blood-urea-nitrogen (BUN) was performed with the ready-to-use Invitrogen Urea Nitrogen (BUN) Colorimetric Detection Kit (Thermo Fisher Scientific) according to the manufacturer’s instructions. Briefly, plasma samples were diluted with aqua dest. 1:20 and mixed with color reagents A and B. After incubation at room temperature for 30 minutes, the coloured product was read at 450 nm.

### Quantitative real-time PCR

RNA was isolated from snap-frozen murine kidneys using RNeasy Mini Kit (Qiagen, Germany) according to the manufacturer’s recommendations, including treatment with DNase (Qiagen) for each sample. RNA was diluted in RNase-free water and the concentration measured using the Invitrogen Qubit Fluorometer (Thermo Fisher Scientific). 500 ng isolated RNA were converted into cDNA with the AffinityScript cDNA Synthesis Kit (Agilent, USA). QPCR was performed using the Brilliant III Ultra-Fast SYBR Green Low ROX qPCR Master Mix (Agilent) with primers listed in Supplementary Table 3. Differences in gene expression were calculated by normalization to the housekeeping gene glucuronidase beta (*Gusb*) and the 2^-ΔΔCT^ method.

### Statistical analysis

For statistical analysis, GraphPad Prism (v. 9.1.2, GraphPad Software, USA) was used. Data are presented as mean (standard error of the mean (SEM)) with individual values. For detection of differences between group means, non-paired one-way analysis of variance (ANOVA) testing was used, assuming a normal distribution and similar variability of the dependent variables. When a significant main effect was found, testing was followed by post-hoc analysis using Sidak’s multiple comparison. The mean of the control group was compared to all fracture groups, and the mean of the vehicle-treated fracture group was compared to AR blocker treatment at the respective timepoints. Values of p < 0.05 were considered statistically significant.

## Results

### ARs addressed by adrenergic blockers are expressed in murine kidneys after femoral fracture

**Fig. 1:**
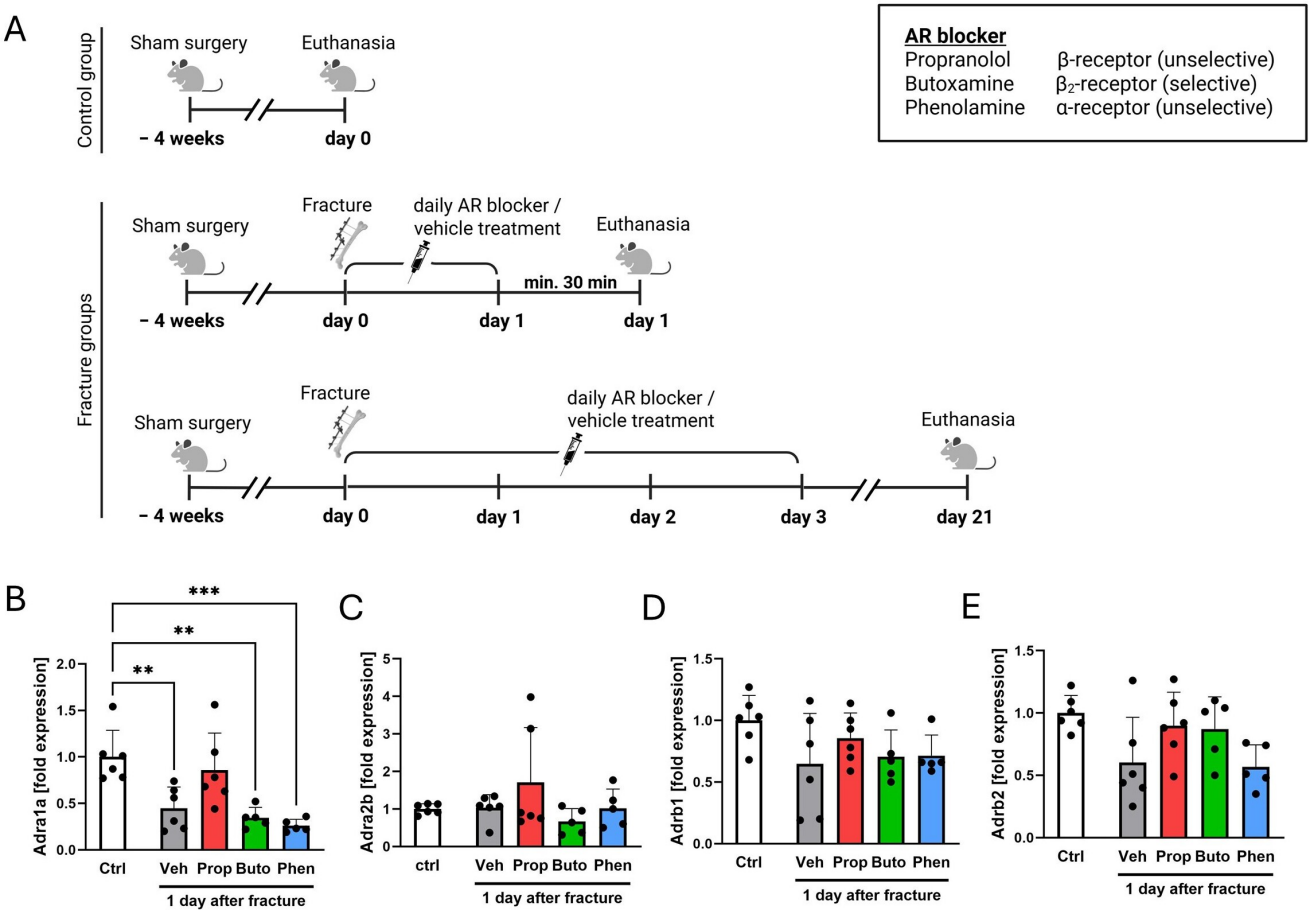
Effects of different adrenoceptor (AR) blockers on AR expression in murine kidneys after fracture. a) Experimental timeline for sham-operated wild-type (WT) mice as control or with femur fracture and AR-blocker/vehicle treatment (created with BioRender.com). Mice underwent sham surgery four weeks prior to a femur fracture, followed by daily AR blocker/vehicle treatment and were euthanized one day or 21 days post fracture. Control mice underwent sham surgery only and were euthanized four weeks later. b) to e) Quantitative real-time polymerase chain reaction revealed significant alterations in respective receptor messenger RNA (mRNA) in whole tissue lysate only for alpha-1a adrenergic receptor (*Adra1a*). N = 5 to 6 per group. *p < 0.05; **p < 0.01; ***p < 0.001. *Adra2b*, alpha-2b adrenergic receptor; *Adrb1*, beta-1 adrenergic receptor; *Adrb2*, beta-2 adrenergic receptor; Buto, butoxamine; Ctrl, control; Phen, phentolamine; Prop, propranolol; Veh, vehicle.

**Fig. 2:**
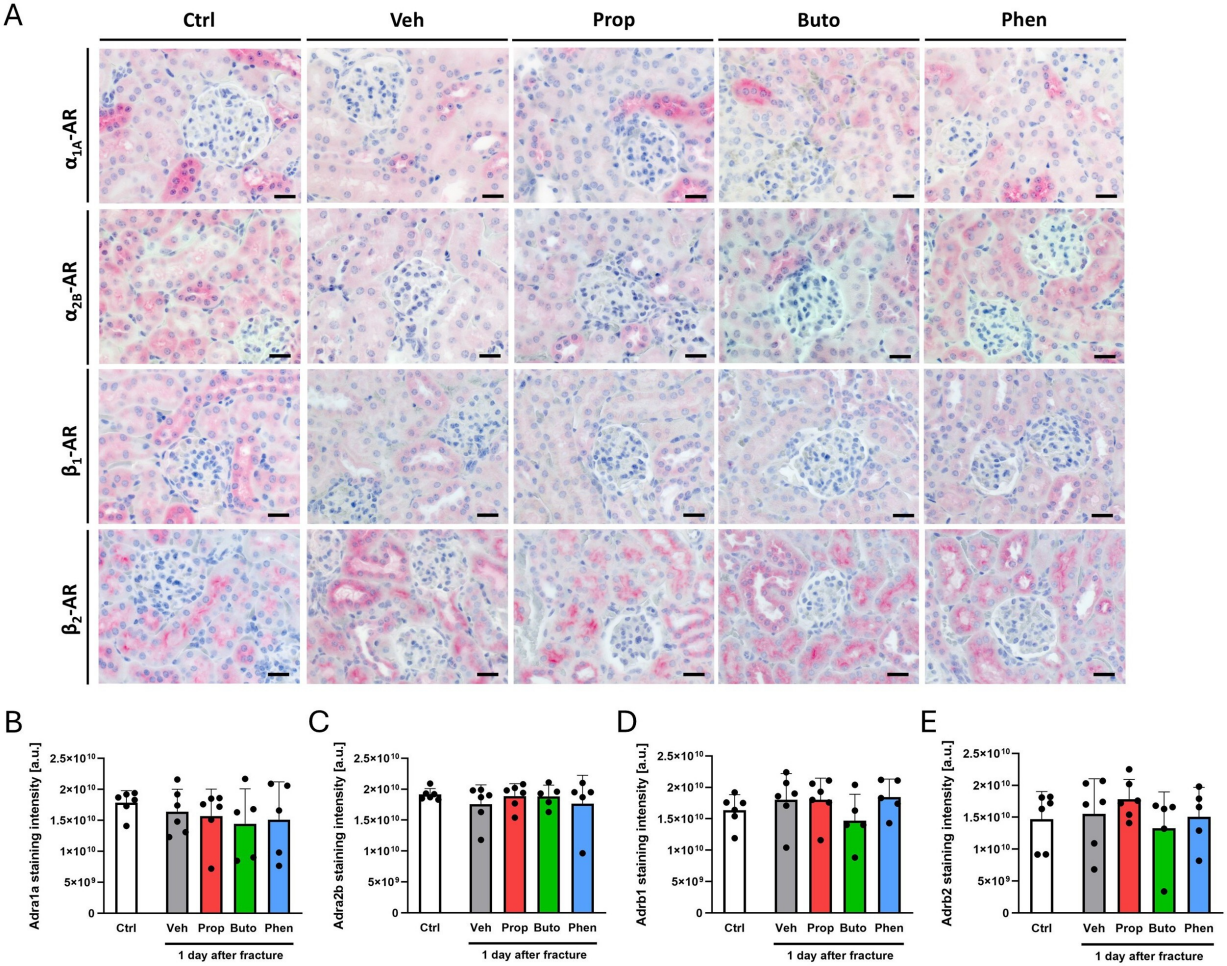
Effects of different adrenoceptor (AR) blockers on AR expression in murine kidneys after fracture. a) Representative images of immunohistochemical (IHC) stained sections of kidney tissue showing the expression of the ARs α1A-AR, α2B-AR, β1-AR, and β2-AR in the kidney (stained in red) one day after fracture and with different receptor blockers. Nuclei were stained with hemalum solution (in blue). Magnification: 400× (bar: 20 μm). N = 3 per group. b) to e) Quantified intensity of red staining in IHC images, performed through measuring the intensity sum red of three visual fields (magnification: 100×) per animal and calculating the mean. N = 5 to 6 per group. Adra1a, alpha-1a adrenergic receptor; Adra2b, alpha-2b adrenergic receptor; Adrb1, beta-1 adrenergic receptor; Adrb2, beta-2 adrenergic receptor; Buto, butoxamine; Ctrl, control; Phen, phentolamine; Prop, propranolol; Veh, vehicle.

The study design is shown in Figure 1a. To analyze the expression of the ARs in the kidney, we tested for two subtypes of the α-AR (α_1A_ and α_2B_), blocked by phentolamine, and the two β-ARs that mediate the response to butoxamine (β_2_) and propranolol (β_1_ and β_2_). The general expression of these receptors on the structures of murine kidneys was initially verified using the CellxGene databank (Supplementary Figure 2)^30^ and confirmed in our animals by gene expression analysis (Figures 1b to 1e) and IHC (Figure 2). We found a significant decrease of the early α_1A_-AR gene expression one day after fracture, for all treatments except propranolol, (Figure 1b), that could not be confirmed on a protein level at this timepoint (Figures 2a and 2b). Expression of the other three receptors could be detected on a gene expression (Figures 1c to 1e) and protein level (Figure 2) and was not altered early after trauma.

### Fracture induces transient AKI regardless of the AR blockade, but does not lead to restriction in function

**Fig. 3:**
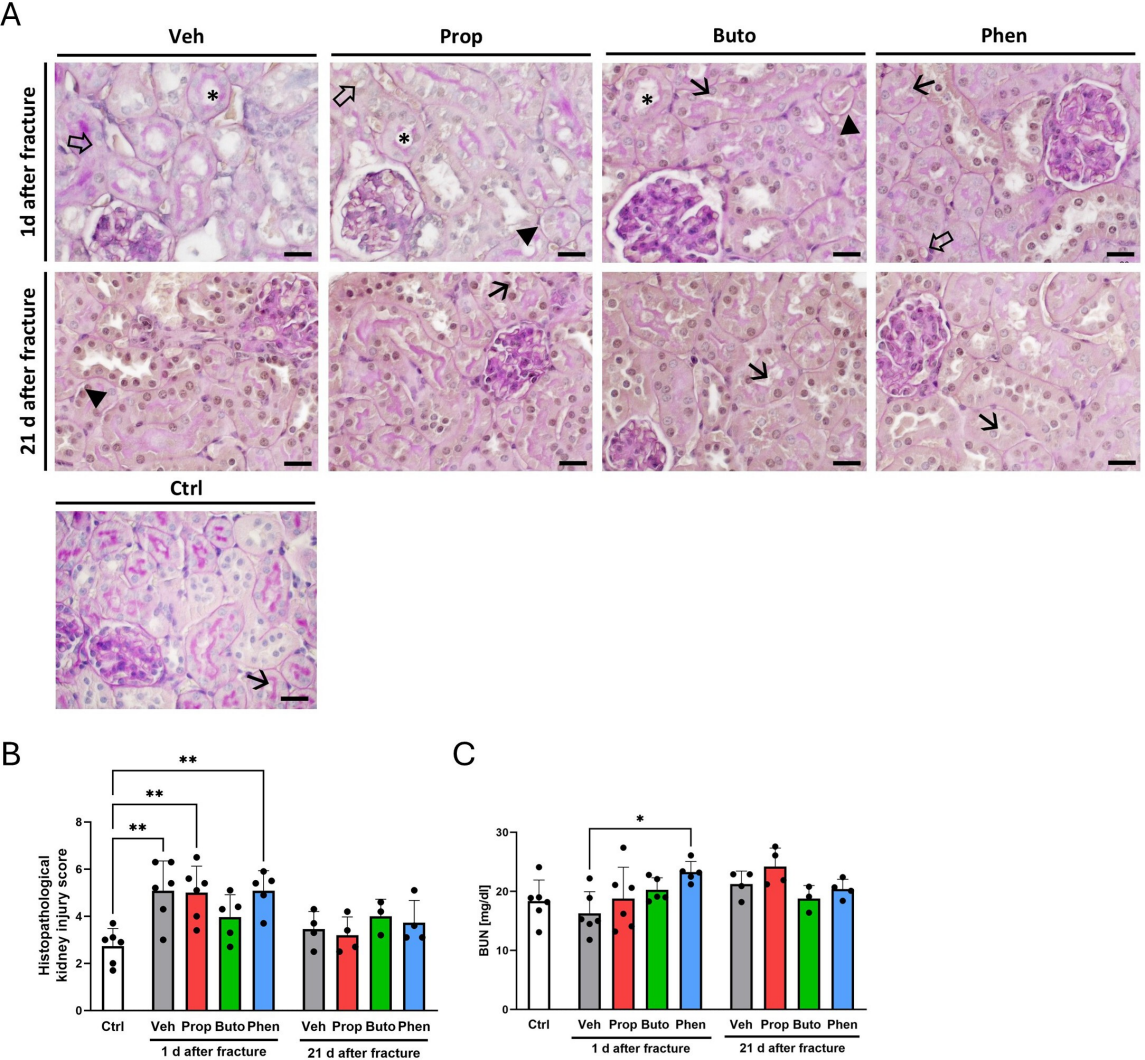
Morphological changes in the kidney following fracture. a) Representative periodic acid-Schiff (PAS) stained sections of kidney tissue, showing histomorphological changes in the proximal tubule after trauma. Different injury criteria were indicated as follows: arrow = loss of brush border, * = tubular dilatation, outlined arrow = vacuolization, black arrowhead = cell sloughing. Magnification: 400x (bar: 20 μm). b) Histomorphological evaluation of kidney damage. Mean injury score was calculated by summing up all mean values of the four criteria from 15 visual fields per animal. c) Blood-urea nitrogen (BUN) one day and 21 days after fracture. N = 3 to 6 for each group. *p < 0.05; **p < 0.01. Ctrl, control; Veh, vehicle; Prop, propranolol; Buto, butoxamine; Phen, phentolamine.

**Fig. 4:**
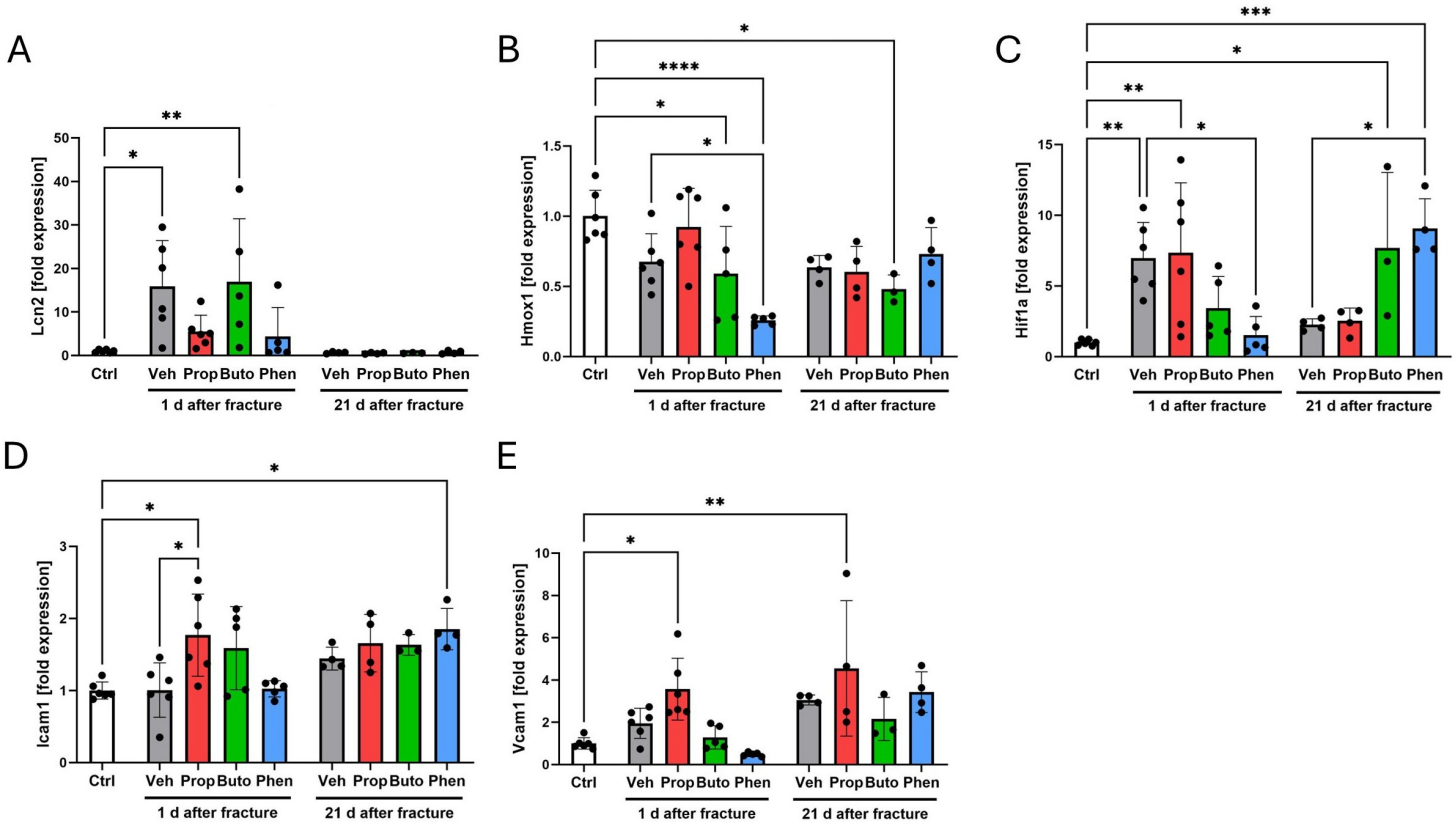
Gene expression analysis from whole kidney lysates via quantitative real-time PCR for a) the kidney injury marker Lipocalin-2 (*Lcn2*), b) heme oxygenase 1 (*Hmox1)*, c) hypoxia-inducible factor 1-alpha (*Hif1a*) and d) intercellular adhesion molecule 1 (*Icam-1*) and e) vascular cell adhesion molecule 1 (*Vcam-1*). N = 3-6 for each group. *p < 0.05; **p< 0.01; ***p < 0.001; ****p < 0.0001. Ctrl, control; Veh, vehicle; Prop, propranolol; Buto, butoxamine; Phen, phentolamine.

To investigate by what means femoral fracture affects the kidney, especially the proximal tubule, we wanted to assess the histopathological acute tubule injury (ATI) by scoring PAS-stained kidney sections according to four distinct criteria: loss of the brush border, cell sloughing, dilation, and vacuolization. The individual scores for each criterion (Supplementary Figure 1) demonstrated a significant increase in brush border loss and tubular dilatation in the fracture group with phentolamine treatment one day post-fracture. Cell sloughing remained unchanged, whereas cell vacuolization was significantly elevated in the fracture groups treated with either vehicle or propranolol. In contrast, butoxamine treatment reduced vacuolization compared to the vehicle-treated fracture group. However, these effects were temporary, since after 21 days all groups returned to their original levels. To facilitate comparative analysis, the four individual scores were combined into a total injury score. Histological signs of kidney injury in the proximal tubule were overall increased one day after fracture compared to the control group, with a statistically significant rise observed in the vehicle-, propranolol-, and phentolamine-treated animals (Figures 3a and 3b). Consistent with the individual scores, no signs of persistent histological damage were detectable 21 days post-fracture. Furthermore, kidney function as determined by BUN was not impaired after fracture (Figure 3c), except for a slight, significant increase in values observed one day after phentolamine treatment compared to the vehicle group. In whole kidney samples, we found significantly higher *Lcn2* gene expression in animals that received vehicle or butoxamine treatment after fracture compared to controls one day post-fracture (Figure 4a). Of note, *Lcn2* encodes neutrophil gelatinase-associated lipocalin (NGAL), a main kidney injury marker that is strongly associated with the development of AKI. Similar to the morphological findings, the expression of the *Lcn2* gene returned to control levels after 21 days. Regarding the kidney’s response to oxidative stress, we observed decreasing expression levels of heme oxygenase 1 (*Hmox1*) one day after fracture, which continued after 21 days (Figure 4b). In contrast, the expression of hypoxia-inducible factor 1-alpha (*Hif1a*) was induced after fracture but not altered by propranolol treatment while the other two blockers (butoxamine and phentolamine) showed a distinct response, with an initial suppression of *Hif1a* expression followed by a significant delayed induction 21 days post-fracture (Figure 4c). Another pathophysiological feature of AKI is the immune response of the kidney, which we addressed by examining the expression of the cell adhesion molecules intercellular adhesion molecule 1 (*Icam-1*) and vascular cell adhesion molecule 1 (*Vcam-1*), both mediators of leucocyte extravasation (Figure 4d and 4e). Propranolol treatment after fracture resulted in an early upregulation of both *Icam1* and *Vcam1* compared to the control group, with *Vcam1* expression remaining elevated even after 21 days. In contrast, phentolamine induced a delayed but significant upregulation of *Icam1* after 21 days, along with a similar trend observed for *Vcam1*.

### Increased proliferation after fracture is reduced by AR blocker treatment

**Fig. 5:**
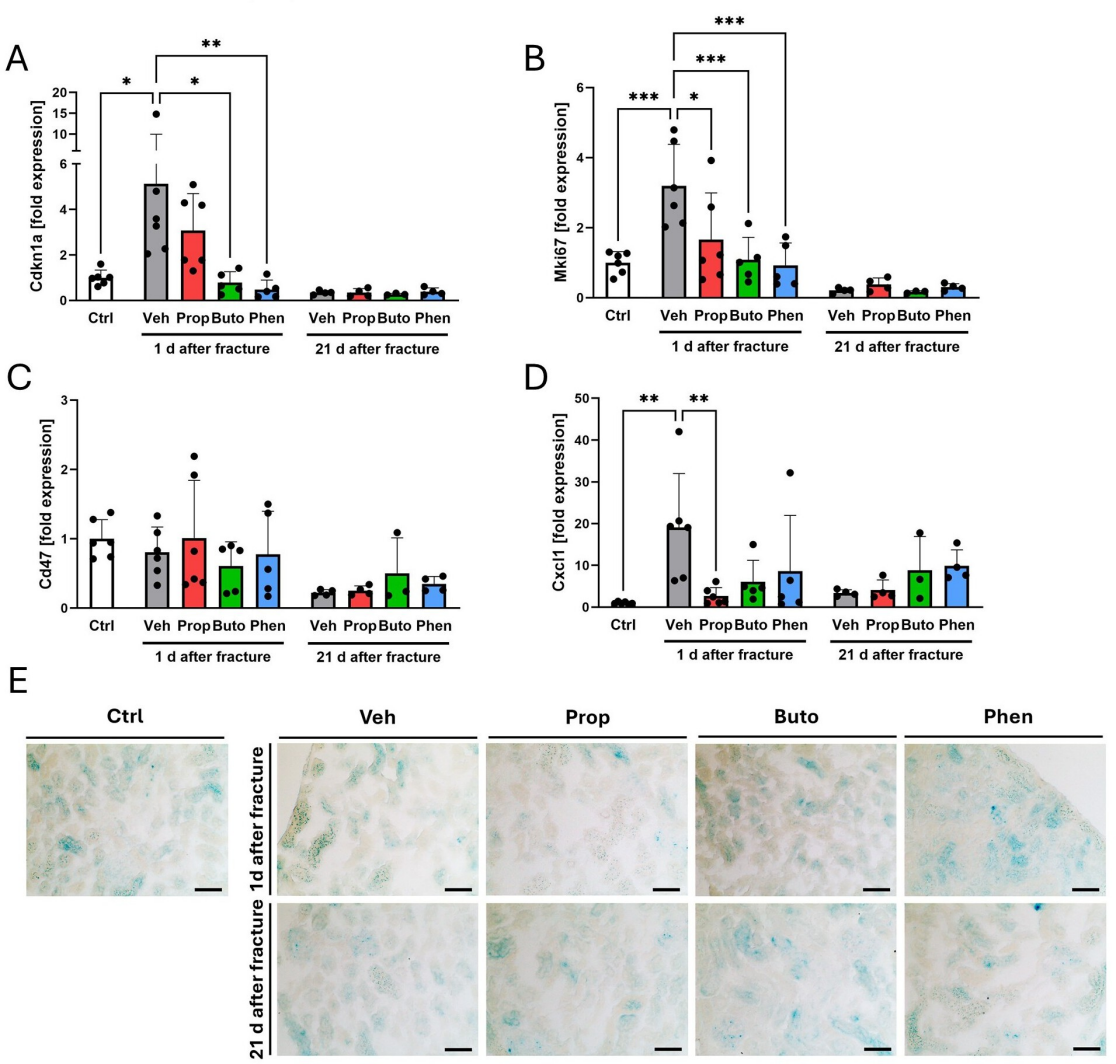
Increased proliferation after fracture can be reduced by adrenergic receptor (AR) blocker treatment and does not cause senescence. Increased proliferation as a consequence of inflammation after fracture was addressed by gene expression analysis from whole kidney lysates performed via quantitative real-time polymerase chain reaction for a) cyclin-dependent kinase inhibitor 1a (*Cdkn1a*), b) antigen identified by monoclonal antibody ki-67 (*Mki67*), c) cluster of differentiation 47 (*Cd47*), and d) C-x-c motif chemokine ligand 1 (*Cxcl1*). N = 3 to 6 per group. The process of cellular senescence was examined using e) senescence-associated beta-galactosidase staining on 8 µm-thick cryosections of the kidney. The presence of senescent cells is indicated by their appearance in blue. Magnification: 200× (bar: 50 μm), N = 5 for control group, N = 2 to 3 for fracture groups. *p < 0.05; **p < 0.01; ***p < 0.001. Buto, butoxamine; Ctrl, control; Phen, phentolamine; Prop, propranolol; Veh, vehicle.

Mice subjected to femoral fracture and vehicle treatment showed significantly elevated expression levels for the proliferation markers cyclin dependent kinase inhibitor 1 a (*Cdkn1a*) (Figure 5a) and antigen identified by monoclonal antibody ki-67 (*Mki67*) (Figure 5b) compared to the control group. After butoxamine or phentolamine treatment, this effect was significantly lower. After 21 days, expression levels were reduced to baseline. Gene expression of cluster of differentiation 47 (*Cd47*) was not altered after fracture compared to the control (Figure 5c). Renal interleukin 1β (*Il1b*) expression was not altered after fracture (Supplementary Figure 3) but we detected significantly increased C-x-c motif chemokine ligand 1 (*Cxcl1*) gene expression compared to the control one day after fracture, which was reduced by AR blocker treatment (Figure 5d).

Since the proliferation and inflammation markers shown in Figures 5a to 5d have also been associated with cellular senescence, we investigated senescence in the kidney tissue using beta-galactosidase staining (Figure 5e). However, no differences in senescence-associated beta-galactosidase staining positivity were observed between the control and fracture groups, as some positive staining was already present at baseline. AR blocker treatments did not notably alter the staining pattern neither one day nor 21 days after fracture.

**Fig. 6:**
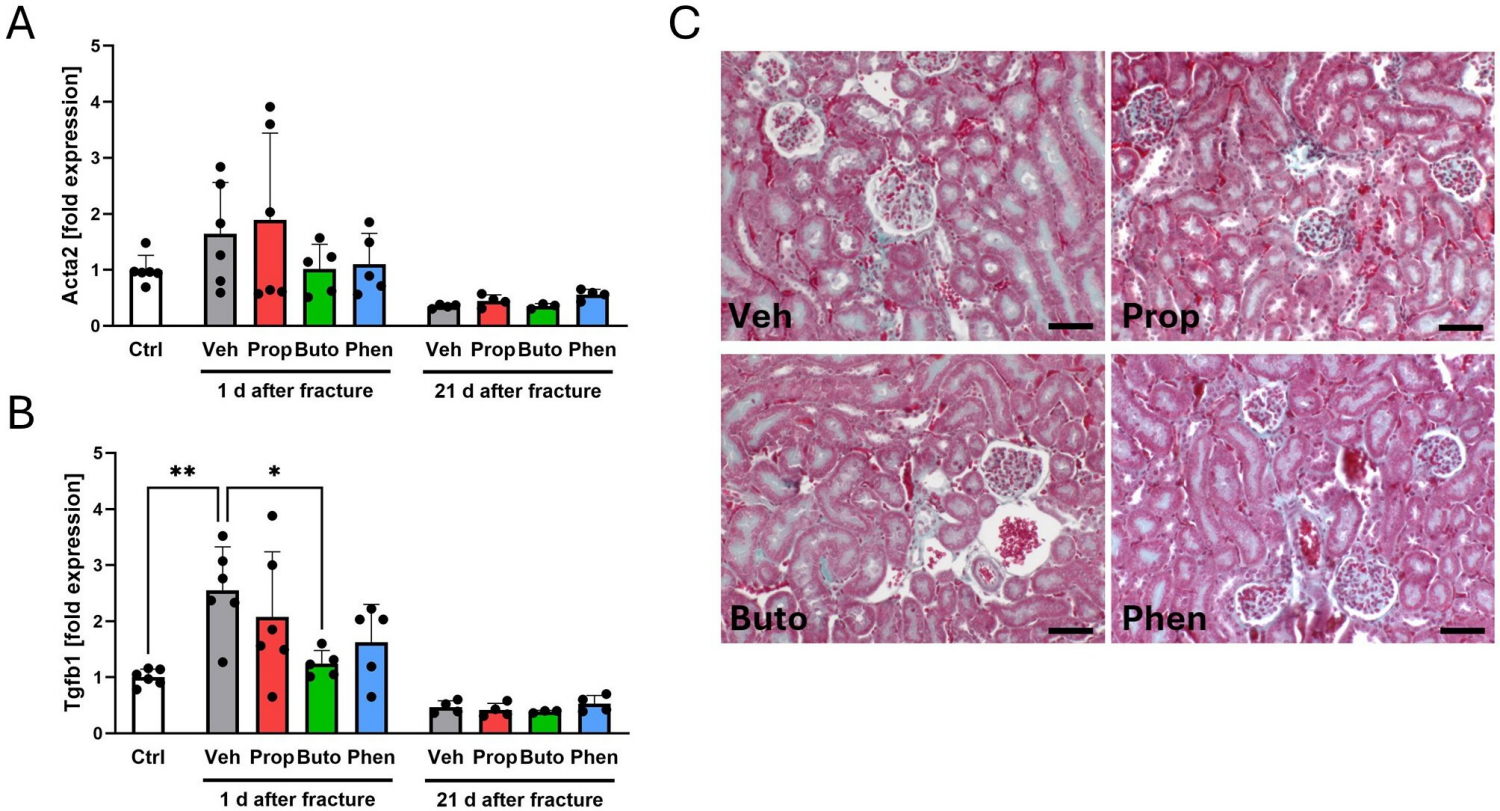
Increased proliferation is not associated with long-term fibrosis. Development of fibrosis in the kidney after fracture was addressed by measuring gene expression of the fibrosis markers a) actin alpha-2 (*Acta2*) and b) transforming growth factor beta-1 (*Tgfb1*) (n = 3 to 6 for each group), as well as through c) representative images of Masson-Goldner stained sections of kidney tissue 21 days post fracture. Nuclei appear dark brown, cytoplasm appears red, erythrocytes appear orange-red, and connective tissue appears green-blue. Magnification: 200× (bar: 50 μm), N = 3 for each group. *p < 0.05; **p < 0.01. Buto, butoxamine; Ctrl, control; Phen, phentolamine; Prop, propranolol; Veh, vehicle.

To determine profibrotic processes which could result from increased proliferation after fracture, marking the transition to chronic kidney injury, we tested the gene expression of the fibrosis markers actin alpha-2 (*Acta2*) and transforming growth factor beta-1 (*Tgfb1*) (Figures 6a and 6b). While expression of *Acta2* was only increased in some animals, *Tgfb1* was significantly upregulated in vehicle-treated animals one day after fracture but not after AR blockade. Moreover, butoxamine treatment significantly reduced *Tgfb1* expression after fracture compared to the vehicle-treated group. However, no histological signs of fibrosis were observed in the kidney 21 days after fracture (Figure 6c).

## Discussion

Femoral fractures belong to the most common fracture types treated in German hospitals,^1,25^ and several studies have shown that there is a connection between bone fracture and an increased risk for the development of TRAKI.^13,31^ The development of post-traumatic kidney injury is the result of a cumulation of various trauma-associated molecular drivers, among them damage-associated molecular patterns (DAMPs), hypoxia, and microcirculation disorders.^15^

Here, we demonstrate that femoral shaft fracture leads to post-traumatic temporal renal injury in female mice. On a microscopic scale, histopathological damage of the proximal tubule could be observed after fracture, according to distinct criteria that have been described for acute tubular injury (ATI).^27-29^ Expression of the gene coding for the early tubular injury marker neutrophil gelatinase-associated lipocalin (NGAL) was significantly increased after fracture,^32^ further indicating damage to the kidney. According to the Kidney Disease: Improving Global Outcomes (KDIGO) criteria,^33^ AKI following femoral fracture surgery manifests within the first 24 hours to seven days. However, in the present study, kidney excretory function was not impaired after fracture according to BUN levels. Measuring the BUN can help to monitor changes in renal function, but can be influenced by various effects like dietary protein intake, catabolism, and tubular reabsorption.^34^ As a limitation of the study, we did not assess potential kidney function impairment using markers such as the glomerular filtration rate (GFR), serum creatinine, or cystatin C.^35^ In response to hypoxia and oxidative stress in the kidney following trauma, we expected increased gene expression of heme oxygenase 1 (*Hmox1*) and hypoxia-inducible factor 1-alpha (*Hif1a*) in the fracture groups. *Hmox1* is naturally upregulated after oxidative stress,^36^ and confers renal protection via anti-inflammatory and immunomodulatory effects.^37^*Hif1a* plays a central role in mediating the adaptive response to hypoxia by inducing the *Hmox1* pathway,^38^ and was also shown to be upregulated during AKI.^39^ However, in our model, *Hmox1* levels were not increased one day after fracture, presumably because significant induction already happened rapidly after injury, with detectable upregulation in as little as a few hours.^38^ Nevertheless, the significantly increased expression of *Hif1a* in the fracture group with vehicle treatment indicates subsequent intrarenal responses due to hypoxia.

In the present study, the immune response in the kidney was not strongly activated after femoral shaft fracture. Leukocyte extravasation, reflected in the gene expression of *Icam-1* and *Vcam-1,*^40^ was not increased in the vehicle-treated group. However, significantly increased expression of C-x-c motif chemokine ligand 1 (*Cxcl1*) one day after fracture could indicate the beginning of increased recruitment of neutrophils.^41^ Up to this point, our findings demonstrate early renal injury after femoral shaft fracture, while also showing renal tissue regeneration by 21 days post-injury, indicating a transient form of AKI. Additional analyses further supported our hypothesis on sufficient regeneration of the kidney, by showing increased levels of the proliferation markers *Cdkn1a* and *Mki67* one day after fracture that normalized 21 days post fracture. Renal tubular epithelial cells initiate proliferation as a part of the repair process after AKI, with the majority of this repair completed within 14 days.^42^ In cases where effective repair in the kidney fails, AKI may progressively result in chronic kidney disease (CKD).^42,43^ Bucaloiu et al^44^ reported that approximately 50% of AKI patients in hospitals who had successfully recovered from AKI were newly diagnosed with CKD during the median follow-up period of 3.3 years. Therefore, we investigated our mouse model for fibrotic alterations, given substantial evidence that injured proximal tubular epithelial cells drive the progression from AKI to CKD by promoting inflammatory and fibrotic responses.^45^*Tgfb1* gene levels were significantly increased one day post fracture, which can also be attributed to its immunoregulatory effects during inflammation,^46^ whereas we found no differential gene expression for *Acta2* post-fracture. After 21 days, neither the fibrosis markers nor histological staining revealed any signs of fibrosis in the kidneys after fracture. The combination of elevated levels of profibrotic factors such as Tgfb1 and proinflammatory mediators like Cxcl1 after fracture may suggest the onset of cellular senescence in the kidney, as both represent components of the senescence-associated secretory phenotype (SASP). SASP is commonly used to assess senescence status, which is triggered by sustained injury and can ultimately lead to tissue fibrosis and organ dysfunction.^47^ However, comparable levels of senescence-associated beta-galactosidase activity in all animals irrespective of group allocation might have resulted from the compromising effects of isoflurane on renal function.^48^

The intrarenal expression of all adrenoceptors relevant to AR-blocker treatment has been previously reported.^22,23,49-51^ The α1-AR is predominantly expressed in arterioles, and the α2-AR is more likely expressed in proximal tubules. Together, they are responsible for stimulating renal vasoconstriction and sodium reabsorption.^22^ Furthermore, the expression of all β-AR subtypes has been identified in all nephron segments.^49^ Kidney β1- and β2-ARs play a key role in regulating renal blood flow, GFR, acid-base balance, sodium and water reabsorption, and renin secretion,^23,50^ whereas the β3-AR is only involved in water reabsorption.^51^ In our study, the presence of the ARs was confirmed through histological staining and qPCR analysis, even after fracture. Therefore, a renal tissue response to all administered AR-blockers could be reasonably assumed. The observed discrepancy between reduced gene expression and unchanged protein levels in the α1-AR staining may reflect temporal differences between transcriptional regulation and detectable changes at the protein level, indicating that the investigated timepoint captured early transcriptional regulation not yet reflected in altered protein levels.

Considering AR-blocker treatment, our findings did not indicate a direct impact on TRAKI but instead point toward systemic effects that are likely to play a contributing role. With the selective β_2_-AR blocker butoxamine, histopathological ATI was not observed after fracture compared to the control group, but *Lcn2* gene expression was significantly increased, reflecting tubular injury. Treatment with the unselective α-AR blocker phentolamine and β-AR blocker propranolol had an opposite effect and could not impede tubular injury but did not lead to increased *Lcn2* levels. This indicates that sympathetic involvement in TRAKI is not only mediated by one AR subtype, but that both α- and β-ARs may play distinct roles. Interestingly, phentolamine seemed to protect the kidney from oxidative stress initially, but ultimately enhanced intrarenal oxidative stress responses. Blockade of α1- and α2- ARs by phentolamine induces vascular smooth muscle relaxation and reduces peripheral resistance.^52^ A well-documented systemic side effect is reflex tachycardia, triggered by excessive vasodilation,^25^ which may lead to secondary local hypoxia. Moreover, NE exhibits a higher affinity for α-ARs than for β-ARs.^53^ Consequently, this may account for the distinct effects observed when blocking ARs by phentolamine versus propranolol or butoxamine. However, the relatively small sample size limited statistical power and the ability to draw firm conclusions; a larger cohort could have provided a more comprehensive understanding. In this study, propranolol did not alter the stress response after fracture but significantly affected the immune response by increasing *Icam-1* and *Vcam-1* levels. Propranolol was shown to have immunomodulatory effects not mediated via β-ARs,^54^ which could account for the lack of similar effects for butoxamine. Furthermore, both butoxamine and phentolamine significantly reduced fracture-induced cellular proliferation compared to the vehicle-treated group, whereas propranolol had only a minor effect. Alongside the reduction of different proinflammatory and profibrotic markers observed with AR-blocker treatment following fracture, these results indicate a renoprotective effect of AR blockade and might reflect a decreased need for tissue repair. This aligns with prior findings that elevated NE levels can exacerbate renal damage by inducing ischaemic injury through vasoconstriction,^55^ and that sympathetic nerve blockade can ameliorate such effects in rodent models.^21^ Whether the protective effects of adrenergic blockers on TRAKI are primarily mediated through systemic mechanisms or direct renal actions remains to be determined. Our results highlight the multifactorial effects of AR blockers on TRAKI, involving a complex interplay of adrenergic pathways and indicating both therapeutic opportunities and challenges. Using another mouse fracture model with higher tissue injury compared to our model, for example a closed fracture model where the muscle is crushed, might also potentiate the effects of the AR blockade on TRAKI, as adrenergic activation might be higher. Additionally, studying older animals could be valuable in the future not only because femoral shaft fractures are being observed more frequently in women after low-energy falls due to osteoporosis,^56,57^ but also considering that especially many older patients are under AR blockade already before experiencing trauma.^24,25^ It must be acknowledged that this work represents a secondary analysis of an ovariectomized model and therefore includes only female animals. Therefore, potential sex-specific differences in the renal response to trauma cannot be excluded, and the applicability of these findings to male animals requires further investigation.

In conclusion, we demonstrate transient AKI in mice following femoral fracture, without impaired long-term kidney function. Oxidative stress and inflammation induced an acute renal response in the early stages following fracture, suggesting a rather mild version of AKI. We found no evidence for a transition from AKI to CKD, as the observed effects were temporary and fibrosis was not indicated. Considering the AR blocker treatment, our findings suggest a role for sympathetic activation in the renal response following trauma, but no sustained protective effects. In particular, the effect of phentolamine-induced α-AR blockade on the kidney’s stress response warrants further investigation, to predict potential harmful effects of adrenergic blockers on the kidney following trauma.

## Data Availability

All data generated or analyzed during this study are included in the published article and/or in the supplementary material.
